# Catamenial Pneumothorax in the Setting of a Recent Stroke

**DOI:** 10.7759/cureus.23860

**Published:** 2022-04-05

**Authors:** Aayush Mittal, Diana Jomaa, Zakaa Hassan, Jennifer Hines, Krishna Thavarajah

**Affiliations:** 1 Internal Medicine, Wayne State University School of Medicine, Detroit, USA; 2 Internal Medicine, Henry Ford Health System, Detroit, USA; 3 Pulmonary and Critical Care Medicine, Legacy Medical Group, Portland, USA; 4 Pulmonary Medicine, Henry Ford Health System, Detroit, USA

**Keywords:** sob - shortness of breath, recurrent pneumothorax, pleural effusion, endometriosis, thoracic endometriosis, catamenial pneumothorax

## Abstract

Catamenial pneumothorax is a unique condition associated with thoracic endometriosis. It often presents in females of reproductive age as a recurrent pneumothorax aligned with the menstrual cycle. We present a case of a young female diagnosed with catamenial pneumothorax within one year of experiencing a stroke. The clinical presentation related to the stroke allowed for a unique diagnostic process and management considerations. The patient was successfully treated with progesterone-based contraception in the setting of an estrogen contraindication.

## Introduction

Catamenial pneumothorax is a rare and often undiagnosed cause of recurrent pneumothorax caused by extra-pelvic involvement of endometriosis [1]. It was previously believed that less than 3-6% of women experiencing spontaneous pneumothorax had an underlying catamenial etiology [2]. However, recent studies have hypothesized that a lack of awareness may have led to frequent misdiagnosis, thus suggesting a higher incidence [3]. In this report, we describe a case of a young female with a history of a stroke who presented for the evaluation of recurrent serosanguinous pleural effusions. We discuss the clinical workup of our patient, as well as key indications that may assist with building a differential diagnosis in similar patient presentations.

## Case presentation

Our patient was a 37-year-old female with a history of middle cerebral artery (MCA) stroke from an embolic mitral valve vegetation nine months prior. She presented to the hospital with five days of generalized fatigue, tachycardia, and dyspnea. Six months prior to this current admission, she had undergone a mitral valve repair procedure and experienced similar symptoms during the postoperative period. She had been noted to have a moderate right-sided pleural effusion that had resolved with the drainage of 500 mL of serosanguinous fluid. At that time, she had been discharged home with plans to complete a course of cardiac rehabilitation.

Two months prior to the current admission, the patient had developed similar symptoms during a rehabilitation appointment. Workup at that time had included a diagnostic echocardiogram, which had demonstrated a pericardial effusion overlying the right atrium and right ventricle. A follow-up CT scan of the chest had indicated a recurrent right pleural effusion resulting in hospital admission for symptom management and further evaluation. Two liters of exudative fluid had been drained via thoracentesis. Subsequent imaging had indicated no residual pleural effusion, pericardial effusion, or endocarditis and the patient had once again been discharged home with plans to continue cardiac rehabilitation.

**Figure 1:**
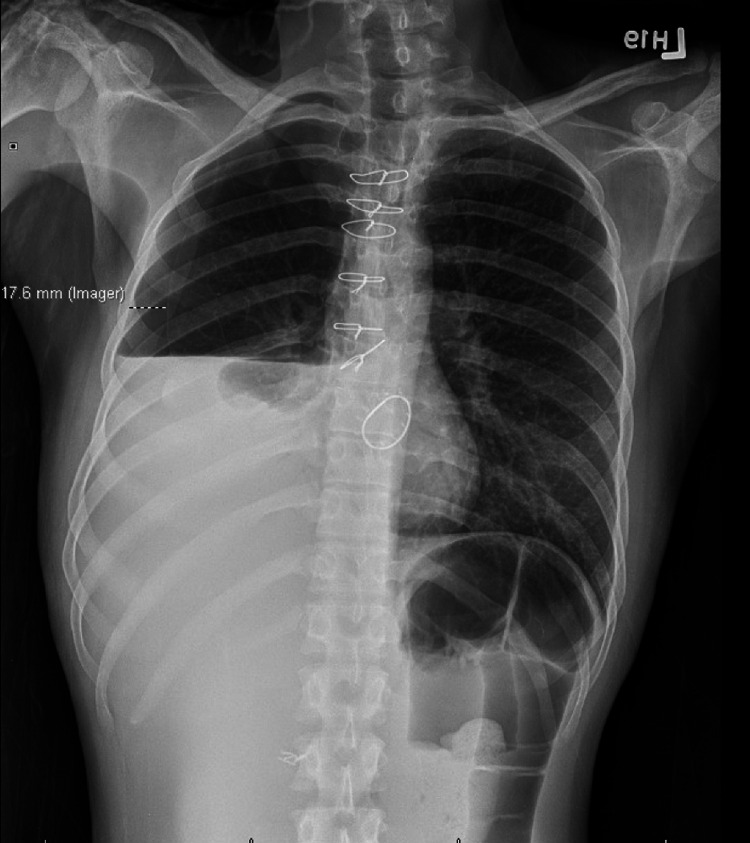
Right pleural effusion on chest X-ray

During the current admission, our patient became concerned after developing similar symptoms of tachycardia and dyspnea. A chest X-ray confirmed that the patient had developed another right pleural effusion (Figure 1) and hydropneumothorax with lateral pleural separation of 17.6 mm. A pigtail catheter was placed in the right pleural space. The pleural fluid analysis demonstrated red color, turbid clarity, protein of 5.2 g/dl, lactate dehydrogenase (LDH) of 223 IU/L, WBC of 1151 with lymphocyte predominance, pH of 7.55, a glucose level of 74 mg/dL, albumin of 2.9 g/dL, and the presence of hemosiderin-like material. This fluid was characterized as exudative as per Light’s Criteria. Anaerobic and aerobic cultures were performed on the fluid, with no growth demonstrated after five days of incubation and with gram stain. We considered a possible autoimmune etiology; however, the autoimmune serology returned negative.

**Figure 2:**
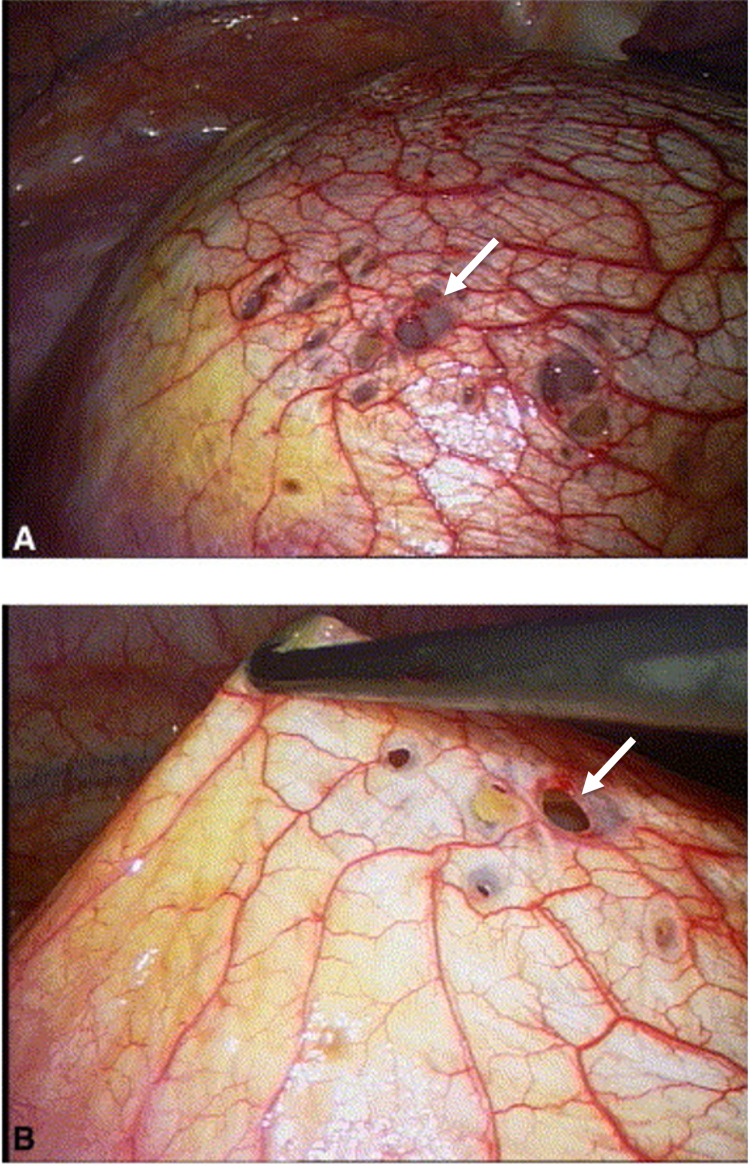
Cyst and fenestrations on the diaphragm (arrows)

At that time, it was still unclear what was causing the patient’s recurrent pleural effusions and spontaneous pneumothorax. Ultimately, the patient underwent a video-assisted thoracic surgery (VATS) with pleurodesis procedure and PleurX catheter placement on admission day five. Surgical findings included a cyst and fenestrations on the diaphragm (Figure 2). Partial resection of the right diaphragm and right pleural biopsy were completed. The pathology report noted chronically inflamed fibroconnective tissue with overlying mesothelium and cystic spaces. Focal features were consistent with endometriosis. Upon further discussion with the patient, she confirmed that these episodes usually occurred within 72 hours of her menstrual cycles, and she endorsed heavier and more painful cycles over the previous year. At that time, given the catamenial etiology, the gynecology service was consulted and they recommended anti-hormonal therapy. Progesterone-only contraception was selected given the prior history of MCA stroke and associated contraindication to estrogen-based therapy.

## Discussion

Thoracic endometriosis was first described in the early 1900s as “a lung tumor which bled every month [4].” With the advancements in diagnostic procedures and pathological capabilities, this definition has been updated. Catamenial pneumothorax, a pneumothorax that occurs within 72 hours of the menstrual period [5], is now recognized as the most common clinical presentation of thoracic endometriosis [6]. Despite these advancements in technology, there are still delays in diagnosis and frequent misdiagnosis of catamenial pneumothorax. One potential reason for this is the non-specific presentation of symptoms. Previous case reports have indicated that these patients are often diagnosed incidentally via endometrial foci or diaphragmatic lesions on imaging [7].

There are many theories about the pathophysiology of thoracic endometriosis. These include retrograde menstruation, metaplasia of coelomic membrane sites, lymphatic or hematologic dissemination, and the prostaglandin theory. The retrograde menstruation theory may explain the unilateral nature of the right diaphragmatic implants in our patient as the left hemidiaphragm obstructs the flow via the falciform and phrenicocolic ligaments [8]. The diaphragmatic lesions observed in our patient were consistent with catamenial pneumothorax; however, these are only noted in 19-33% of patients [9]. Treatment often involves pleurodesis and hormonal therapy; however, recurrence may occur if the primary foci of endometriosis are not removed.

As our patient had a past history of a cerebrovascular accident (CVA), estrogen-based hormonal therapy was contraindicated. Pleurodesis was completed during the VATS procedure, but pleurodesis alone is associated with a high failure rate [10]. Hence, our patient was considered for progesterone-only contraception. The administration of progestins impacts the frequency of the pulsatile GnRH release, thereby causing diminished pituitary secretion of gonadotropins and ovarian secretion of steroid hormones. However, if the patient does not respond to progesterone-based pharmacotherapy, a GnRH agonist should be considered. Although GnRH agonists similarly impact the pulsatile GnRH, they have been shown to have a greater effect on endometrial tissue atrophy and estradiol suppression. New agents such as GnRH antagonists are also being developed [11]. We believe that this presentation of catamenial pneumothorax with a potential relationship to a CVA is a rare and elusive diagnosis. Although we remain uncertain of the pathology of the mitral valve vegetations found in our patient, the relationship with thoracic endometriosis and subsequent treatment must be considered.

## Conclusions

Based on previous literature, we believe that thoracic endometriosis and catamenial pneumothorax should be included in the differential diagnosis for women with recurrent pleural effusions and pneumothorax. A thorough review of gynecologic history and review of systems should be completed to avoid delays in diagnosis, especially in women with symptoms during the perimenstrual time period. The pathophysiology can be confirmed via biopsy during a VATS procedure with pleurodesis. We hope that increased awareness of this condition will enable early intervention and diagnosis in these patients. Treatment typically involves surgical intervention and hormonal therapy, with certain patients having a recurrence of symptoms despite this intervention. We presented a case of a woman with a prior history of mitral valve vegetations causing a CVA, leading to a contraindication of estrogen-based therapy. Further research is required to explore the relationship between endometriosis and stroke. However, we hope that this case provides greater insight into a unique clinical presentation and treatment options after surgical intervention.
